# 1,25-hydroxyvitamin D3 decreases endoplasmic reticulum stress-induced inflammatory response in mammary epithelial cells

**DOI:** 10.1371/journal.pone.0228945

**Published:** 2020-02-10

**Authors:** Gaiping Wen, Klaus Eder, Robert Ringseis

**Affiliations:** Institute of Animal Nutrition and Nutrition Physiology, Justus-Liebig-University Giessen, Giessen, Germany; University of Illinois, UNITED STATES

## Abstract

Recent studies indicated that intramammary administration of active vitamin D_3_ hormone (1,25D_3_) inhibits the inflammatory process associated with mastitis. We hypothesized that attenuation of endoplasmic reticulum (ER) stress by 1,25D_3_ in mammary epithelial cells (MECs) is an important cellular mechanism contributing to this beneficial effect of intramammary treatment with 1,25D_3_. To test this hypothesis, the effect of 1,25D_3_ was studied on induction of ER stress in a transformed human MEC line, MCF-7 cells. Treatment with two different ER stress inducers, thapsigargin (TG) and tunicamycin (TM), caused a dose-dependent induction of ER stress as evident from up-regulation of protein kinase RNA-like ER kinase (*PERK*), heat shock protein family A (Hsp70) member 5 (*HSPA5*), activating transcription factor (*ATF4*), *ATF6*, DNA damage inducible transcript 3 (*DDIT3*) and spliced X-box binding protein 1 (*sXBP1*) and impaired cell viability and decreased expression of vitamin D receptor (*VDR*) in MCF-7 cells (*P* < 0.05). Treatment with 1,25D_3_ (100 nM) inhibited TG (10 nM)- and TM (1 μg/mL)-induced mRNA and/or protein levels of *ATF4*, *ATF6*, *DDIT3* and *HSPA5* in MCF-7 cells (*P* < 0.05). In addition, 1,25D_3_ (100 nM) antagonized the effect of TG (10 nM) and TM (1 μg/mL) on mRNA and protein levels of *VDR* and mRNA levels of genes involved in production and degradation of 1,25D_3_ in MCF-7 cells (*P* < 0.05). Moreover, 1,25D_3_ (100 nM) inhibited nuclear factor-κB (NF-κB) activation in response to TM (10 nM) and TG (1 μg/mL) in MCF-7 cells. In conclusion, the present findings show that 1,25D_3_ is effective in attenuating ER stress and the NF-κB-driven inflammatory response in MCF-7 cells. This indicates that attenuation of ER stress by 1,25D_3_ in MECs may contribute to the recently observed inhibitory effect of intramammary treatment of dairy cows with 1,25D_3_ on the inflammatory process associated with mastitis.

## Introduction

Mastitis refers to an inflammation of mammary tissue mostly caused by infections with different pathogenic bacteria. Mastitis in dairy cattle has great economic impact due to milk production loss, costs for veterinary treatment and potentially fatal outcome [1]. Despite antibiotics are widely used for the treatment of mastitis [2], this treatment strategy is increasingly considered critically due to limited effectiveness owing to the occurrence of antibiotic-resistant strains. Against this background, dietary interventions may be a reasonable strategy in the prevention and treatment of the inflammatory response associated with mastitis.

Previous *in vitro*-studies revealed that the active vitamin D_3_ hormone, 1,25-hydroxyvitamin D_3_ (1,25D_3_), improves bactericidal capacity of human and bovine monocytes against common bacterial pathogens involved in mastitis development [3–5]. This effect likely contributes to a decreased bacterial growth in mammary glands experimentally infected with *Streptococcus uberis* [6] and an increased expression of host-defense genes in mammary immune cells [7, 8] of dairy cows subjected to intramammary treatment with 1,25D_3_ or its metabolite 25D_3_. Apart from monocytes and lymphocytes, mammary epithelial cells (MECs) surrounding alveoli in the milk parenchyma in the mammary gland act as important innate immunocompetent cells by producing a significant amount of pro-inflammatory cytokines upon pathogen contact [9–11]. Pathogen-dependent induction of immune functions in MECs is mediated by toll-like receptor (TLR2, TLR4)-dependent sensing of pathogen-associated molecular patterns (PAMPs) like lipopolysaccharide (LPS) and lipoteichoic acid [12]. Sensing of PAMPs leads to the activation of the key regulator of inflammation nuclear factor-kappa B (NF-κB), thereby, stimulating the production of pro-inflammatory cytokines, chemokines, reactive oxygen species (ROS) and other host-defense molecules *via* inducing more than hundred immune relevant genes [13]. Despite the NF-κB-regulated acute inflammatory response is important to effectively combat the infectious bacteria causing mastitis, it is important that the inflammatory process is rapidly attenuated because prolonged production of ROS, cytokines and other inflammatory molecules causes structural damage of the mammary gland through injurious action on cellular components (lipids, proteins, DNA), thereby, decreasing cell viability, and ultimately inducing cell death [14].

Recently, it was shown in a mouse model of mastitis that the inflammatory process induced by LPS administration in the mammary gland is also closely related to induction of endoplasmic reticulum (ER) stress in mammary tissue and that attenuation of ER stress by a secondary plant metabolite protects from LPS-induced mastitis by inhibiting the pro-inflammatory NF-κB signaling pathway [15]. Thus, inhibition of ER stress is likely a suitable strategy in the prevention and therapy of the inflammatory process associated with mastitis. ER stress describes a state characterized by the accumulation of misfolded proteins owing to an imbalance in ER quality control pathways, such as folding, trafficking and degradation [16]. As a consequence of ER stress, the unfolded protein response (UPR), which involves three different transmembrane ER stress sensors, namely ATF6 (activating transcription factor 6), IRE1 (inositol-requiring protein 1a) and PERK (protein kinase RNA-like ER kinase), is initiated in order to restore normal ER function by different mechanisms including transient attenuation of new protein synthesis, stimulation of IRE1-dependent mRNA degradation and induction of molecular chaperones [17–19]. The close link between NF-κB-driven inflammation and ER stress during mastitis is likely explained by their mutual interaction; activation of all ER stress sensors causes downstream activation of NF-κB, and ER stress-inducing stimuli, such as ROS and cytokines, are produced from immunocompetent cells during the course of the inflammatory process [20, 21].

Owing to their high metabolic and secretory capacity, MECs are particularly susceptible to environmental conditions that cause ER stress and thus the UPR pathway is critical in maintaining ER homeostasis in MECs [22–24]. Interestingly, several indications exist that 1,25D_3_ inhibits ER stress in different cell types [25, 26]. Whether this is also the case in MECs remains to be established. In light of the above-described beneficial effects of 1,25D_3_ on mechanisms involved in mastitis development, we hypothesized that attenuation of ER stress by 1,25D_3_ in MECs is an important cellular mechanism leading to inhibition of NF-κB-driven inflammation. To test this hypothesis, the effect of 1,25D_3_ was studied on induction of ER stress in a transformed human MEC line. Despite ER stress is known to be indirectly induced by several inflammatory mediators like LPS [27], ER stress was induced in the present study by more selective ER stress inducers, such as tunicamycin (TM), which causes ER stress by inhibiting protein glycosylation [28], and thapsigargin (TG), which causes ER stress by inhibiting the sarco-/endoplasmic reticulum calcium ATPase [29]. Because 1,25D_3_ exerts many of its effects *via* the vitamin D receptor (VDR) [30] and ER stress was found to suppress transcriptional activity of the VDR gene in human epithelial cells [31], the effect of ER stress and 1,25D_3_ on expression of *VDR* and of genes encoding hydroxylases involved in local 1,25D_3_ production and degradation was also studied.

## Materials and methods

### Chemicals

1,25D_3_ and tunicamycin (TM) were purchased from Sigma-Aldrich (Steinheim, Germany) and thapsigargin (TG) was purchased from Biomol (Hamburg Germany). From all test compounds, stock solutions in dimethylsulfoxide (DMSO; Sigma-Aldrich) were prepared (1,25D_3_: 10 mM; TM: 5 mg/mL; TG: 5 mM).

### Cell culture

MCF-7 cells were obtained from Cell Lines Service (Eppelheim, Germany) and grown in Dulbecco´s Modified Eagle Medium (DMEM) supplemented with 10% fetal bovine serum (FBS; both from Gibco/Life Technologies, Darmstadt, Germany) and 0.05 mg/mL gentamicin (Invitrogen, Karlsruhe) in a cell incubator at 37°C in a humidified atmosphere of 95% air and 5% CO_2_. Growth medium was changed every 2 days. After reaching a confluence of 70–80%, the cells were either sub-cultivated or used for experiments. Aliquots from stock solutions were directly added to the growth medium and control cells were incubated with the same vehicle concentration (DMSO) at the concentrations indicated in figure legends. All experiments were performed at three times from a different cell passage number (= independent experiments).

### Cell viability assay

The 3-(4,5-dimethylthiazol-2-yl)-2,5-diphenyltetrazolium bromide (MTT; Sigma-Aldrich, Steinheim, Germany) assay was used to assess cell viability in response to TM, TG and 1,25D_3_. For the MTT assay, MCF-7 cells were seeded in 96-well culture plates at a density of 1.2 x 10^4^ cells per well and incubated as indicated in figure legends. The MTT assay was carried out exactly as described recently [32].

### RNA isolation and quantitative real-time polymerase-chain reaction (qPCR) analysis

For qPCR analysis, MCF-7 cells were seeded in 24-well culture plates at a cell density of 6 x 10^4^ per well and incubated as indicated in figure legends. Total RNA extraction, cDNA synthesis and qPCR were performed as described recently [32]. Gene-specific primers were synthesized by Eurofins MWG Operon (Ebersberg, Germany). Characteristics of primers are listed in **Table 1**. Normalization was carried out using multiple reference genes (*ATP5B*, *GAPDH*, *SDHA*, *YWHAZ*) as described recently [32].

**Table 1:** Sequences of gene-specific primers used for qPCR analysis.

Gene name	Primer sequence (forward, reverse)	Product size (bp)	NCBI GenBank
*Reference genes*			
*ATP5B*	TCGCGTGCCATTGCTGAGCT CGTGCACGGGACACGGTCAA	218	NM_001686
*GAPDH*	GCCTTCCGTGTCCCCACTGC CAATGCCAGCCCCAGCGTCA	211	XR_002046
*SDHA*	CCAAGCCCATCCAGGGGCAAC TCCAGAGTGACCTTCCCAGTGCCAA	100	NM_004168
*YWHAZ*	TGGGGACTACGACGTCCCTCAA CATATCGCTCAGCCTGCTCGG	115	NM_003406
*Target genes*			
*ATF4*	GTTCTCCAGCGACAAGGCT GCATCCAAGTCGAACTCCTT	150	NM_001675
*ATF6*	GTCTCCCCTTTCCTTATATGG AAGGCTTGGGCTGAATTGAAG	164	NM_007348
*CEBPA*	GTGGACAAGAACAGCAACGAG CATTGTCACTGGTCAGCTCCA	133	NM_001287435
*CYP2R1*	CTCAGTGGGTGAACTCATCAT CGTACAACTGCATCTTCAGAG	264	NM_024514
*CYP24A1*	GCTTGTATCGACAACCGGTT CAGACAGGCTTTTAAATACGG	294	NM_000782
*CYP27B1*	GTTTGCATTTGCTCAGAGGCA GCTCATACAGAGCCCAAGAG	218	NM_000785
*DDIT3*	GGAGAACCAGGAAACGGAAAC GCTTGAGCCGTTCATTCTCTT	129	NM_001195053
*FASN*	GGCCGTGGTCTTGAGAGATG TAGTTGCTCTGTCCCGCATTG	189	NM_004104
*HMGCR*	GACAGGATGCAGCACAGAATG TTGAACACCTAGCATCTGCAAAC	179	NM_000859
*HSPA5*	GAGGAGGAGGACAAGAAGGA CAGGAGTGAAGGCGACATAG	157	NM_005347
*LDLR*	GTCAGCTCCACAGCCGTAAG CCCAGAGCTTGGTGAGACATTG	128	NM_000527
*NFKB1*	GCAGATGGCCCATACCTTCAA CACCATGTCCTTGGGTCCAG	285	NM_003998
*PERK*	GTCGCCAATGGGATAGTGACG GCTCTCGTTTCCATGTCTGG	166	NM_004836
*RXRA*	TTCGCTAAGCTCTTGCTC ATAAGGAAGGTGTCAATGGG	113	NM_0012319020
*VDR*	CCAGTTCGTGTGAATGATGG GTCGTCCATGGTGAAGGA	384	NM_000376
*sXBP1*	TGCTGAGTCCGCAGCAGGTG GCTGGCAGGCTCTGGGGAAG	169	NM_005080

### Immunoblotting

For immunoblotting, MCF-7 cells were seeded in 6-well culture plates at a cell density of 1.8 x 10^5^ per well and treated as indicated in figure legends. For detection of HSPA5 and DDIT3 cell lysates were prepared with radioimmunoprecipitation (RIPA) assay lysis buffer [50 mM Tris (pH 7.5), 150 mM NaCl, 1 mM EDTA, 1% Triton X-100, 0.1% SDS, and 1% sodium deoxycholate] containing protease inhibitor cocktail (Sigma-Aldrich). For detection of phosphorylated NF-κB (p-NF-κB), cell lysates were prepared with RIPA lysis buffer containing the phosphatase inhibitors sodium fluoride (5 mM) and sodium-orthovanadat (1 mM) (both from Sigma-Aldrich) and protease inhibitor cocktail. For detection of VDR and NF-κB nuclear extracts were prepared with the Nuclear Extract Kit (Active Motif, La Hulpe, Belgium) according to the manufacturer’s protocol. Protein concentrations of lysates and nuclear extracts were determined by the BCA protein assay kit (Interchim, Montlucon, France) and BSA as standard. A total amount of 10–15 μg protein was separated by SDS-PAGE and electrotransferred to nitrocellulose membranes. The membranes were incubated with primary antibodies [rabbit anti-HSPA5 (dilution 1:5000) and mouse anti-DDIT3 (dilution 1:2000) (both from Thermo Fisher Scientific, Darmstadt, Germany), rabbit anti-VDR (dilution 1:300), rabbit anti-NF-κB (dilution 1:500) and mouse anti-p-NF-κB (dilution 1:500; all from Santa Cruz, Heidelberg, Germany)] at 4°C overnight. The primary antibodies against mouse anti-β-actin (dilution 1:40.000, Abcam, Cambridge, UK) and rabbit anti-vinculin (dilution 1:10.000, Thermo Fisher Scientific) were incubated as reference proteins for normalization at room temperature for 2 h. The membranes were washed and then incubated with horseradish peroxidase-conjugated secondary antibodies anti-rabbit-IgG (dilution 1:10.000, Sigma-Aldrich) and anti-mouse-IgG (dilution 1:10.000, Santa Cruz) at room temperature for 2 h. Afterward, blots were developed using enhanced chemiluminescence (ECL) Plus (GE Healthcare, München, Germany). The signal intensities of specific bands were detected with a Bio-Imaging system (Syngene, Cambridge, UK) and quantified using Syngene GeneTools software (nonlinear dynamics; Syngene). For calculation of protein levels, the band intensity of the target protein was normalized by that of the reference protein.

### Statistical analysis

All data represent means and SD. The means and SD were calculated from all replicates for the same treatments of all independent experiments. In each independent experiment, all treatments were represented in 1–8 wells (= technical replicates per treatment: immunoblotting, one; qPCR, three; MTT assay, eight) depending on the plate format. Statistical analysis was performed using the Minitab statistical software (Rel. 13.0, State College, PA, USA). Data from qPCR and MTT assay were subjected to 2-factorial ANOVA with classification factors being treatment (T), experiment (E) and the interaction of both factors (T x E). Because data from immunoblotting included only one replicate per treatment within each independent experiment, treatment effects were analyzed by 1-factorial ANOVA. For statistically significant F values, individual means of the treatment groups were compared by Fisher´s multiple range test. Effects were considered significant if *P* < 0.05.

## Results and discussion

### Treatment with ER stress inducers cause induction of ER stress-induced UPR and an impairment of cell viability in MCF-7 cells

**Fig 1:**
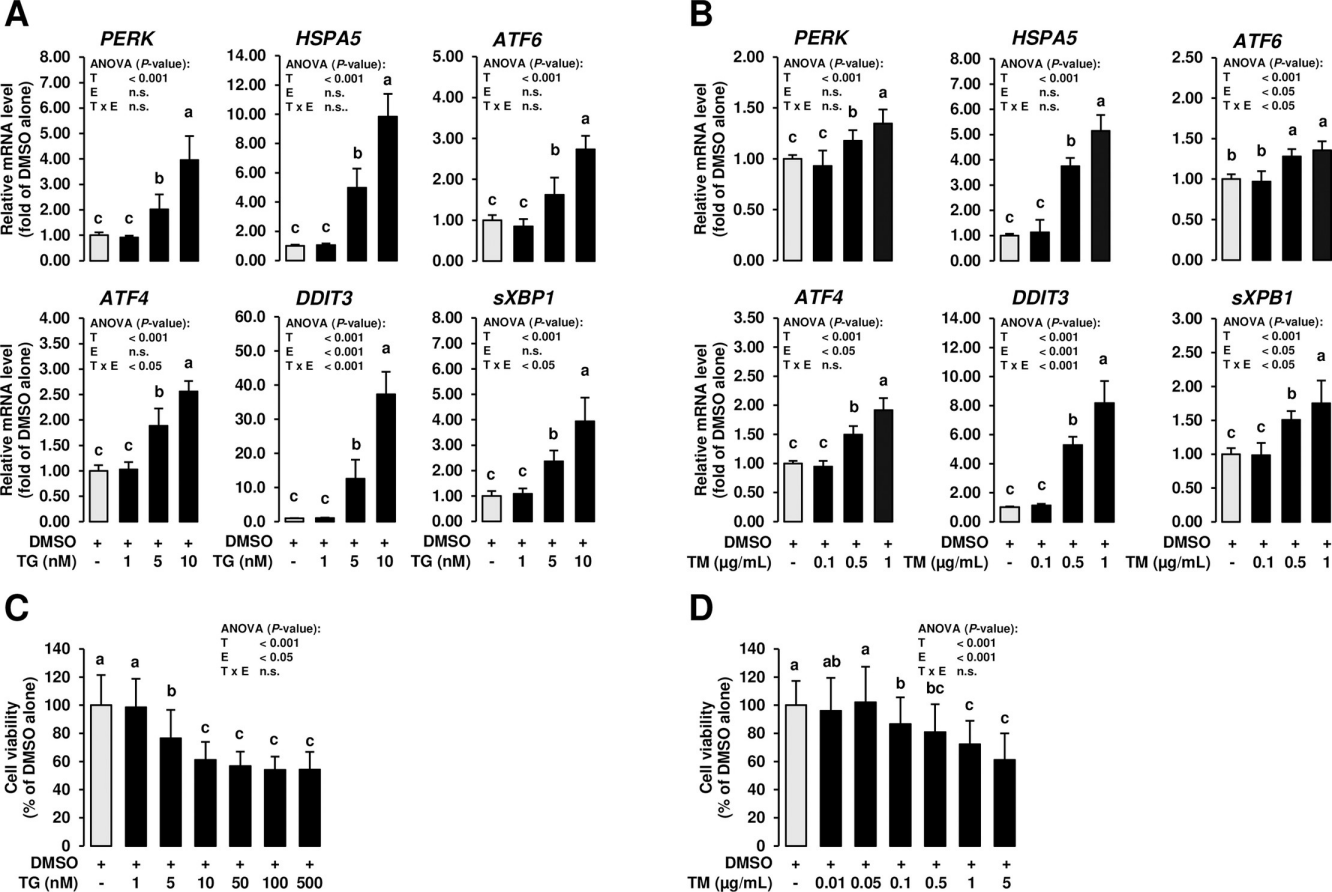
Effect of ER stress inducers on expression of UPR target genes and cell viability in mammary epithelial cells. MCF-7 cells were incubated in DMEM with 1% FBS with either vehicle alone (DMSO, 0.1% v/v) or increasing concentrations of TG (dissolved in DMSO; A: 1 to 10 nM; C: 1 to 500 nM) or TM (dissolved in DMSO; B: 0.1 to 1 μg/mL; D: 0.01 to 5 μg/mL) for 24 h. A, B: Bars represent relative mRNA levels expressed as fold of vehicle alone and are means ± SD from three independent experiments. C, D: Bars represent relative cell viability expressed as percent of vehicle alone and are means ± SD from three independent experiments. A-D: Bars with unlike letters are significantly different (*P* < 0.05). 2-factorial ANOVA classification factors: treatment (T), experiment (E), interaction (T x E). Abbreviations: ATF4/6, activating transcription factor 4; DDIT3, DNA damage inducible transcript 3; HSPA5, heat shock protein family A (Hsp70) member 5; PERK, protein kinase RNA-like ER kinase; sXBP1, spliced X-box binding protein 1.

To investigate the induction of ER stress in MCF-7 cells, MCF-7 cells were treated for 24 h with TG and TM at different concentrations (TG: 1, 5 and 10 nM; TM: 0.1, 0.5 and 1 μg/mL), which were recently reported to induce ER stress in other cell types [33, 34]. Despite TG and TM induce ER stress *via* distinct mechanisms [28, 29], both ER stress inducers caused a quite similar response in MCF-7 cells. Treatment with TG and TM dose-dependently induced ER stress as evident from marked upregulation of known ER stress-sensitive genes such as *PERK*, heat shock protein family A (Hsp70) member 5 (*HSPA5*), *ATF6*, *ATF4*, DNA damage inducible transcript 3 (*DDIT3*) and spliced X-box binding protein 1 (*sXBP1*) (*P* < 0.05; **Fig 1A and 1B**) at 5 and 10 nM TG and 0.5 and 1 μg/mL TM. In line with the fact that sustained ER stress causes cell death *via* apoptosis, 24 h-treatment with both ER stress inducers impaired cell viability at concentrations ≥ 5 nM TG (*P* < 0.05; **Fig 1C**) and ≥ 0.1 μg/mL TM (*P* < 0.05; **Fig 1D**). At TG concentrations of 5 and 10 nmol/L, MCF-7 cell viability was reduced by 23 and 39%, respectively, while TM concentrations of 0.1 and 1 μg/mL decreased cell viability by 13 and 28%, respectively.

### Treatment with 1,25D_3_ inhibits ER stress induced by TM and TG in MCF-7 cells

**Fig 2:**
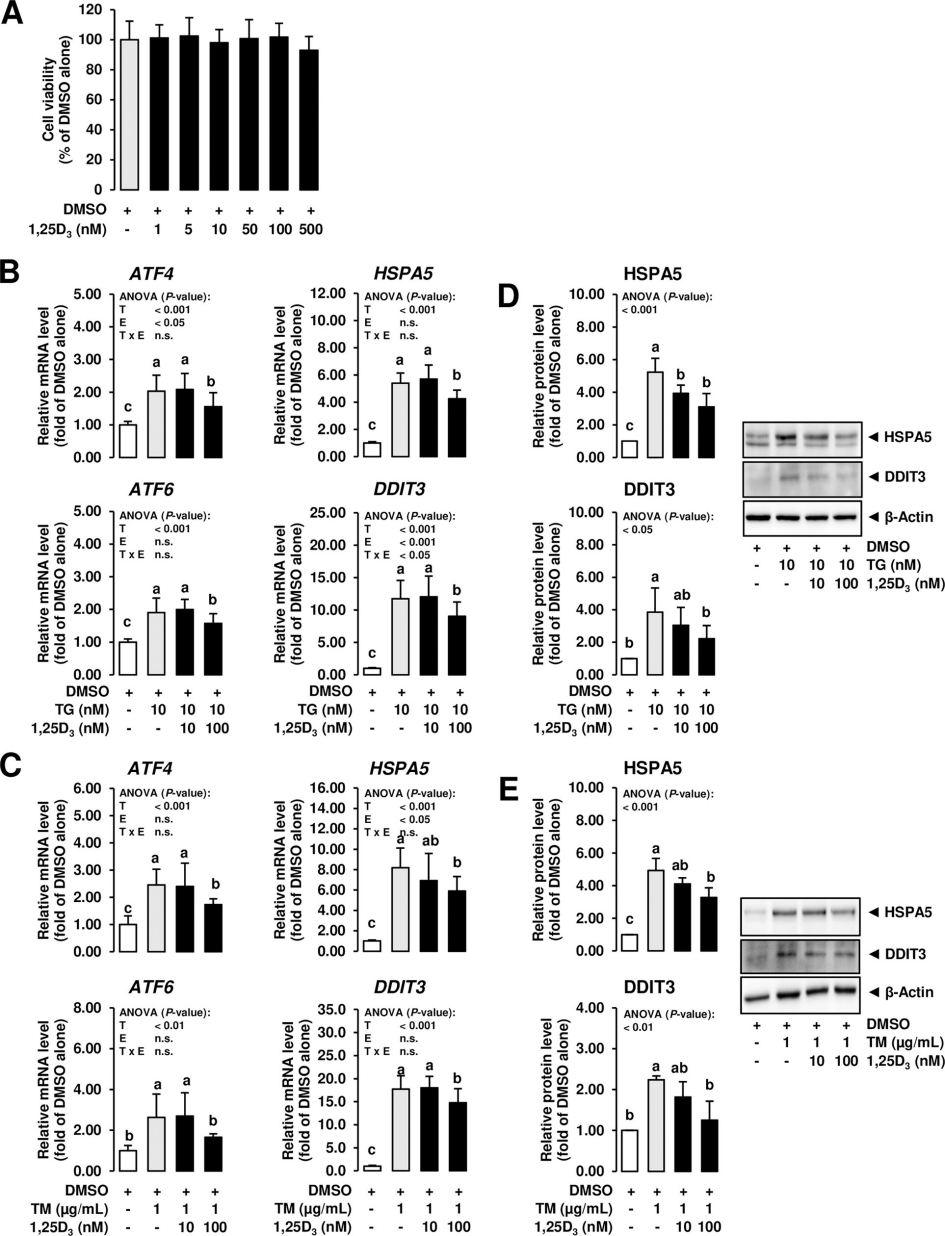
Effect of 1,25D_3_ on ER stress-induced expression of UPR target genes in mammary epithelial cells. A: MCF-7 cells were incubated in DMEM with 1% FBS with either vehicle alone (DMSO, 0.1% v/v) or increasing concentrations of 1,25D_3_ (dissolved in DMSO; 1 to 500 nM) for 24 h. Bars represent relative cell viability expressed as percent of vehicle alone and are means ± SD from three independent experiments. B, C: MCF-7 cells were pre-incubated in DMEM with 1% FBS and with either vehicle alone (DMSO, 0.1% v/v) or 1,25D_3_ (10 or 100 nM) alone for 24 h and subsequently co-incubated in DMEM with 1% FBS and with either vehicle alone (DMSO, 0.1% v/v), TG (10 nm) or TM (1 mg/mL) alone or 1,25D_3_ (10 or 100 nM) together with TG (10 nM) or TM (1 μg/mL) for additional 24 h. Bars represent relative mRNA levels (B, C) and relative protein levels (D, E) expressed as fold of vehicle alone and are means ± SD from three independent experiments. A-E: Bars with unlike letters are significantly different (*P* < 0.05). 2-factorial ANOVA classification factors: treatment (T), experiment (E), interaction (T x E). Abbreviations: ATF4/6, activating transcription factor 4; DDIT3, DNA damage inducible transcript 3; HSPA5, heat shock protein family A (Hsp70) member 5.

In order to investigate the potential of 1,25D_3_ to modulate ER stress induced by ER stress inducers in MCF-7 cells, the ER stress inducers were used at 10 nM (TG) and 1 μg/mL (TM). At these concentrations of TG and TM, ER stress was clearly induced but cell viability was only moderately impaired thus enabling us to avoid strong bias of impaired cell viability on cellular effects caused by ER stress. Treatment of MCF-7 cells with 1,25D_3_ alone at a wide concentration range (1 to 500 nmol/L), which has been shown to exert biological effects in other cell culture studies [35], for 24 h did not impair cell viability compared to vehicle control cells (**Fig 2A**). Thus, the effect of 1,25D_3_ on ER stress signaling induced by TG and TM was studied at a low (10 nmol/L) and a high (100 nmol/L) concentration. In this experiment, cells treated with 1,25D_3_ and ER stress inducers were pre-treated for 24 h with 1,25D_3_ alone and subsequently co-treated for 24 h with 1,25D_3_ and ER stress inducers. As shown in **Fig 2B and 2C**, ER stress induced by TG (10 nM) and TM (1 μg/ml) was attenuated by the high concentration (100 nmol/L) of 1,25D_3_ as evident from decreased mRNA levels of the ER stress-sensitive genes *ATF4*, *HSPA5*, *ATF6*, *DDIT3*, *PERK* and *sXBP1* (the latter two are not shown; *P* < 0.05). In addition, treatment with 100 nmol/L 1,25D_3_ attenuated TG- and TM-induced protein levels of the ER stress markers *HSPA5* and *DDIT3* in MCF-7 cells (*P* < 0.05; **Fig 2D and 2E**). These results clearly indicated that 1,25D_3_ inhibits ER stress induction by TG and TM in MCF-7 cells.

### Treatment with 1,25D_3_ antagonizes the effect of ER stress inducers on expression of VDR in MCF-7 cells

**Fig 3:**
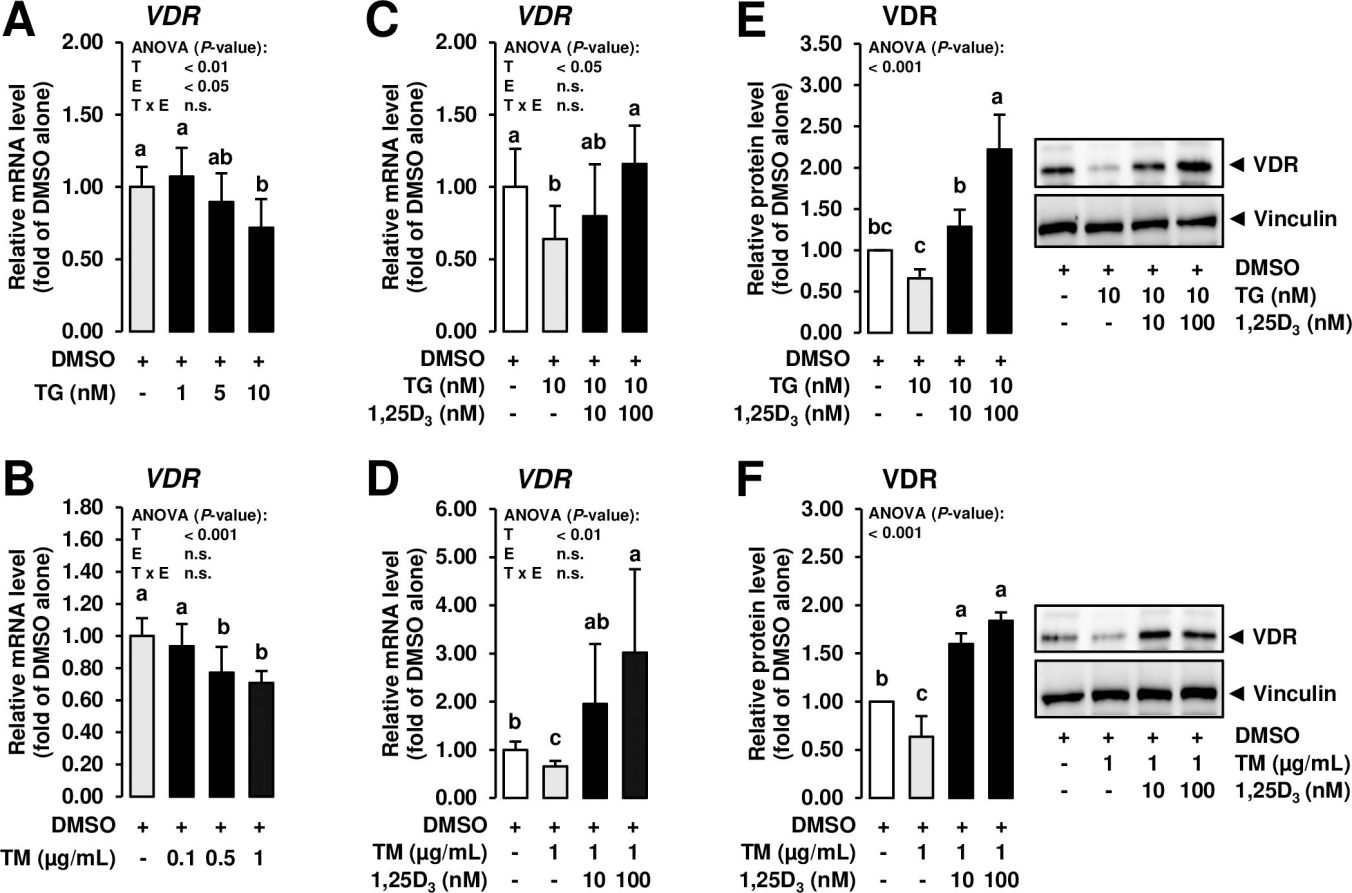
Effect of ER stress inducers alone and combined effect of ER stress inducers and 1,25D_3_ on expression of VDR in mammary epithelial cells. A, B: MCF-7 cells were incubated in DMEM with 1% FBS with either vehicle alone (DMSO, 0.1% v/v) or increasing concentrations of TG (dissolved in DMSO; 1 to 10 nM) or TM (dissolved in DMSO; 0.1 to 1 μg/mL) for 24 h. C-F: MCF-7 cells were pre-incubated in DMEM with 1% FBS and with either vehicle alone (DMSO, 0.1% v/v) or 1,25D_3_ (10 or 100 nM) alone for 24 h and subsequently co-incubated in DMEM with 1% FBS and with either vehicle alone (DMSO, 0.1% v/v), TG (10 nm) or TM (1 mg/mL) alone or 1,25D_3_ (10 or 100 nM) together with TG (10 nM) or TM (1 μg/mL) for additional 24 h. Bars represent relative mRNA levels (A, B, C, D) and relative protein levels (E, F) expressed as fold of vehicle alone and are means ± SD from three independent experiments. A-H: Bars with unlike letters are significantly different (*P* < 0.05). 2-factorial ANOVA classification factors: treatment (T), experiment (E), interaction (T x E). Abbreviations: VDR, vitamin D receptor.

Apart from non-genomic effects, most biological effects of 1,25D_3_ are mediated by the nuclear VDR which is present in many cell types including MCF-7 cells [36, 37]. In cultured human keratinocytes, treatment with TG was found to decrease transactivation of the *VDR* gene [31] indicating that the response of epithelial cells to 1,25D_3_ is impaired under conditions of ER stress. In addition, siRNA-mediated knockdown of *VDR* was reported to inhibit the ability of 1,25D_3_ to repress ER stress in human umbilical vein endothelial cells [26]. This indicated that inhibition of ER stress by 1,25D_3_ is VDR-dependent in endothelial cells and that this mechanism may also play a role in the inhibition of ER stress in MCF-7 cells. To clarify if ER stress affects expression of *VDR* in MCF-7 cells, the effect of increasing concentrations of TG and TM were investigated on the mRNA level of *VDR*. Treatment with TG and TM decreased *VDR* mRNA level at a concentration ≥ 10 nM and ≥ 0.5 μg/mL, respectively (*P* < 0.05; **Fig 3A and 3B**), which clearly indicates that *VDR* expression is decreased in the presence of ER stress in this MEC line—an observation which is in contrast to the effect of ER stress in an immortalized kidney tubule cell line [33].

It has long been known that *VDR* expression is regulated by 1,25D_3_ at least in the intestine, i.e., 1,25D_3_ increases *VDR* mRNA level and newly synthesized VDR [38]. Thus, to next study whether treatment with 1,25D_3_ is capable of increasing *VDR* expression in MCF-7 cells exposed to ER stress, the expression of *VDR* was studied at both the mRNA and the protein level in MCF-7 cells treated with both ER stress inducers. While treatment of MCF-7 cells with TG (10 nM) and TM (1 μg/mL) decreased mRNA and protein levels of *VDR* compared to vehicle control cells (*P* < 0.05), treatment with 1,25D_3_ of MCF-7 cells co-incubated with TG and TM dose-dependently increased mRNA and protein levels of *VDR* above levels of vehicle control cells (*P* < 0.05; **Fig 3C–3F**). This finding indicated that 1,25D_3_ counter-regulates the inhibitory effect of ER stress on *VDR* expression in MCF-7 cells.

**Fig 4:**
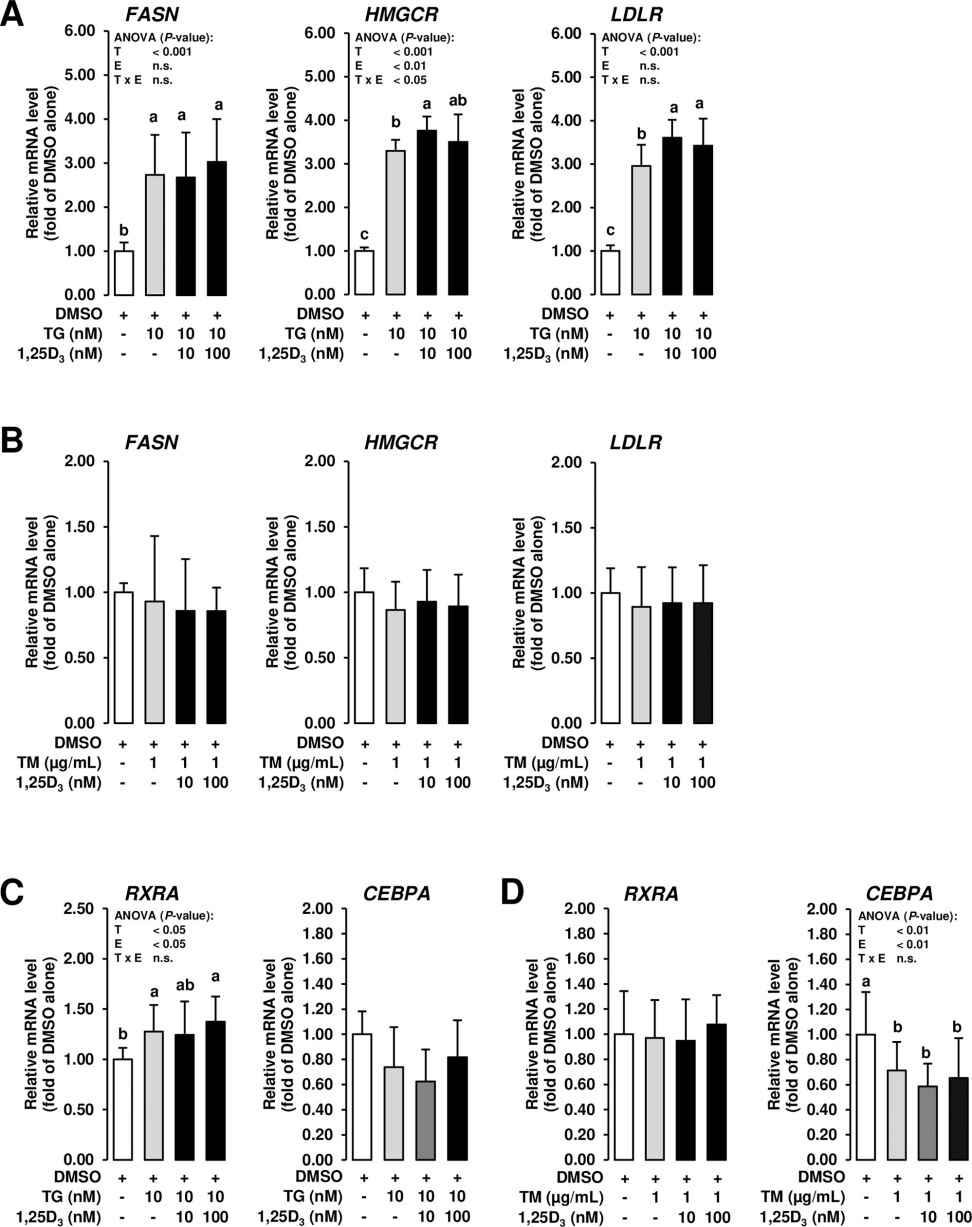
Effect of ER stress inducers alone and combined effect of ER stress inducers and 1,25D_3_ on expression of genes involved in lipid synthesis and transcriptional regulators of VDR in mammary epithelial cells. MCF-7 cells were pre-incubated in DMEM with 1% FBS and with either vehicle alone (DMSO, 0.1% v/v) or 1,25D_3_ (10 or 100 nM) alone for 24 h and subsequently co-incubated in DMEM with 1% FBS and with either vehicle alone (DMSO, 0.1% v/v), TG (10 nm; A, C) or TM (1 mg/mL; B, D) alone or 1,25D_3_ (10 or 100 nM) together with TG (10 nM; A, C) or TM (1 μg/mL; B, D) for additional 24 h. Bars represent relative mRNA levels expressed as fold of vehicle alone and are means ± SD from three independent experiments. Bars with unlike letters are significantly different (*P* < 0.05). 2-factorial ANOVA classification factors: treatment (T), experiment (E), interaction (T x E). Abbreviations: CEBPA, CCAAT enhancer binding protein alpha; FASN, fatty acid synthase; HMGCR, 3-hydroxy-3-methylglutaryl-CoA reductase; LDLR, low density lipoprotein receptor; RXRA, retinoid X receptor alpha.

Attenuation of new protein synthesis by PERK-dependent phosphorylation/inactivation of eukaryotic initiation factor 2α and IRE1-dependent decay of mRNAs during ER stress are important mechanisms of the UPR which decreases the load of ER folding and degradation pathways, thereby, allowing the ER to better cope with misfolded proteins accumulating during ER stress [18]. It is thus possible that this mechanism is responsible for the downregulation of *VDR* in MCF-7 cells. In cultured adipocytes, which are highly specialized cells for the synthesis and storage of neutral lipids, it has been found that lipogenesis, which takes place in the ER, and expression of lipogenic genes is decreased by treatment with TG and TM [39], likely as a result of adaptive attenuation of new protein synthesis during ER stress. *De novo*-synthesis of lipids, such as fatty acids and cholesterol, is also an important metabolic function of MECs, thereby, providing sufficient amounts of lipids to be secreted into the milk. In order to evaluate if the expression of lipogenic and cholesterogenic genes is also reduced in MCF-7 cells under conditions of ER stress as a consequence of an impaired metabolic capacity of the ER, the mRNA levels of key lipogenic and cholesterogenic genes, fatty acid synthase (*FASN*), 3-hydroxy-3-methylglutaryl-CoA reductase (*HMGCR*) and low density lipoprotein receptor (*LDLR*), were determined. Unlike *VDR* gene expression, gene expression of *FASN*, *HMGCR* and *LDLR* was not reduced in MCF-7 cells treated with ER stress inducers (**Fig 4A and 4B**), which might indicate that the metabolic capacity of the ER was not severely impaired by ER stress induction. The observation that treatment with TG even induced *FASN*, *HMGCR* and *LDLR* in MCF-7 cells is in line with findings in hepatocytes, where TG and TM were reported to stimulate proteolytic activation of the master regulator of lipid synthesis sterol regulatory element-binding protein-1 (SREBP-1) *via* splicing of XBP1 [40–42]. This ER stress-dependent mechanism aims to provide lipids required to facilitate expansion of the ER during the UPR. The disparate regulation of genes involved in lipid synthesis in response to ER stress in adipocytes [39] and MCF-7 is hard to explain from our data, but it shows that the decrease of *VDR* expression in response to TG and TM in MCF-7 cells cannot be simply attributed to an attenuation of global protein synthesis. It is well-known that regulation of human and mouse *VDR* gene expression is exceedingly complex and is mediated by multiple enhancers located both upstream of the *VDR* gene transcription start site and within downstream enhancers, all of which contain multiple binding sites for different transcription factors, including RUNX family transcription factor 2, CCAAT/enhancer binding protein (CEBP), cAMP response element binding protein and even retinoid X receptor alpha (RXRA) [43–46], which is known as the heterodimerization partner of VDR [47]. The contribution of one or more of these transcription factors to the basal expression of the VDR gene might be critical considering recent observations that siRNA-mediated suppression of these transcription factors reduced the basal level of *VDR* gene expression [45]. Thus, it is not unlikely that the induction of ER stress in MCF-7 cells may have decreased, by whatever mechanism, the expression of one or more of these transcription factors required for the transcription of the *VDR* gene. To address this issue, we have determined the expression of two of these transcription factors, *RXRA* and *CEBPA*. While the mRNA level of *RXRA* was not decreased by treatment of MCF-7 cells with either TG or TM, the mRNA level of *CEBPA* was decreased or tended (*P* < 0.1) to be decreased in MCF-7 cells treated with TM and TG, respectively (**Fig 4C and 4D**). However, treatment with 1,25D_3_ failed to counter-regulate the inhibitory effect of ER stress on *CEBPA* expression. Thus, these data may help explain the down-regulation of *VDR* gene transcription in MCF-7 cells exposed to ER stress, but cannot provide an explanation for the counter-regulatory role of 1,25D_3_. Future studies are warranted to clarify this issue.

### Treatment with 1,25D_3_ antagonizes the effect of ER stress inducers on expression of genes involved in production and degradation of 1,25D_3_ in MCF-7 cells

**Fig 5:**
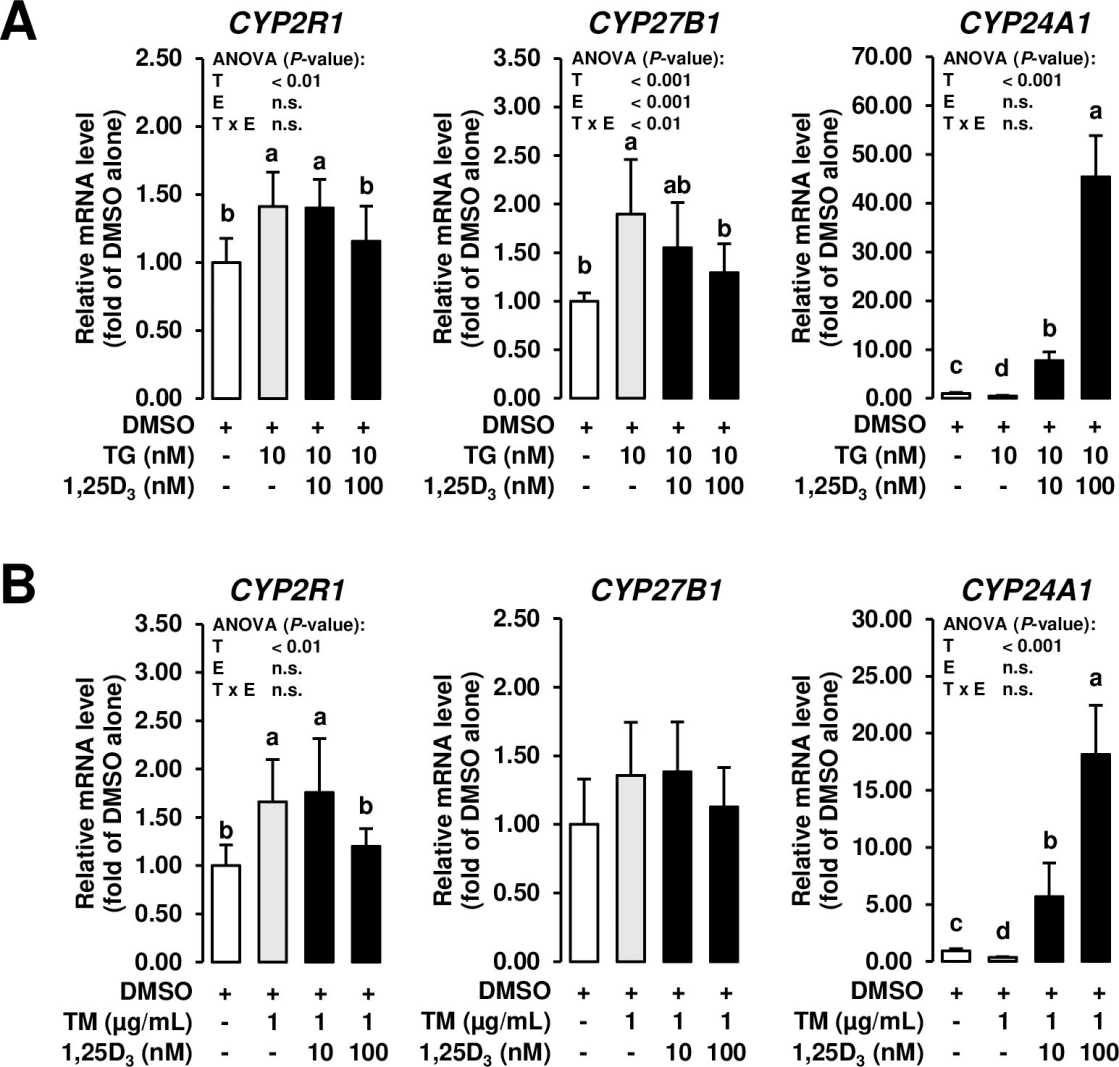
Effect of ER stress inducers alone and combined effect of ER stress inducers and 1,25D_3_ on expression of genes involved in production and degradation of 1,25D_3_ in mammary epithelial cells. MCF-7 cells were pre-incubated in DMEM with 1% FBS and with either vehicle alone (DMSO, 0.1% v/v) or 1,25D_3_ (10 or 100 nM) alone for 24 h and subsequently co-incubated in DMEM with 1% FBS and with either vehicle alone (DMSO, 0.1% v/v), TG (10 nm; A) or TM (1 mg/mL; B) alone or 1,25D_3_ (10 or 100 nM) together with TG (10 nM; A) or TM (1 μg/mL; B) for additional 24 h. Bars represent relative mRNA levels expressed as fold of vehicle alone and are means ± SD from three independent experiments. Bars with unlike letters are significantly different (*P* < 0.05). 2-factorial ANOVA classification factors: treatment (T), experiment (E), interaction (T x E). Abbreviations: CYP2R1, cytochrome P450 family 2 subfamily R member 1; CYP24A1, cytochrome P450 family 24 subfamily A member 1; CYP27B1, cytochrome P450 family 27 subfamily B member 1.

Because intramammary levels of 1,25D_3_ are known to be affected by mammary epithelial cell production and degradation of 1,25D_3_ *via* specific hydroxylases, the effect of ER stress inducers on the mRNA levels of 25-hydroxylase (encoded by *CYP2R1*), 1α-hydroxylase (encoded by *CYP27B1*), and 24-hydroxylase (encoded by *CYP24A1*) was also studied. While the mRNA levels of *CYP2R1* and *CYP27B1*, both of which are involved in stepwise hydroxylation of vitamin D_3_ into 1,25D_3_, were increased by TG and TM alone compared to vehicle control cells, the mRNA levels of these genes were decreased by the high concentration of 1,25D_3_ in MCF-7 cells co-incubated with ER stress inducers to levels observed in vehicle control cells (*P* < 0.05; **Fig 5A and 5B**). At least *CYP27B1* expression has been recently demonstrated to be upregulated in monocytes and macrophages in response to TLR activation [3]. This mechanism likely explains upregulation of *CYP27B1* mRNA in innate immune cells of the bovine mammary gland during mastitis [48], because mastitis-inducing bacteria are sensed by TLRs. Since TLR activation is also known to induce ER stress, upregulation of *CYP27B1* might be also indicative of ER stress induction and the observed downregulation of *CYP27B1* in MCF-7 cells treated with 1,25D_3_ might indicate that ER stress was attenuated in MCF-7 cells. Apart from this, it is also possible that the cellular 1,25D_3_ status of MCF-7 cells was impaired due to ER stress induction and the decreased mRNA levels of *CYP27B1* and *CYP2R1* in response to 1,25D_3_ supplementation reflect a feedback regulatory mechanism signaling sufficient cellular 1,25D_3_ levels. Supportive of such a feedback regulatory mechanism is also the observation that the mRNA level of *CYP24A1*, which catalyzes the breakdown of 1,25D_3_ and which is known to be highly upregulated by 1,25D_3_ [49, 50], was decreased by treatment of MCF-7 cells with both ER stress inducers, whereas *CYP24A1* mRNA level was strongly increased by 1,25D_3_ in a dose-dependent manner in MCF-7 cells co-incubated with TG and TM (*P* < 0.05; **Fig 5A and 5B**).

### Treatment with 1,25D_3_ inhibits NF-κB activation in response to TM and TG in MCF-7 cells

**Fig 6:**
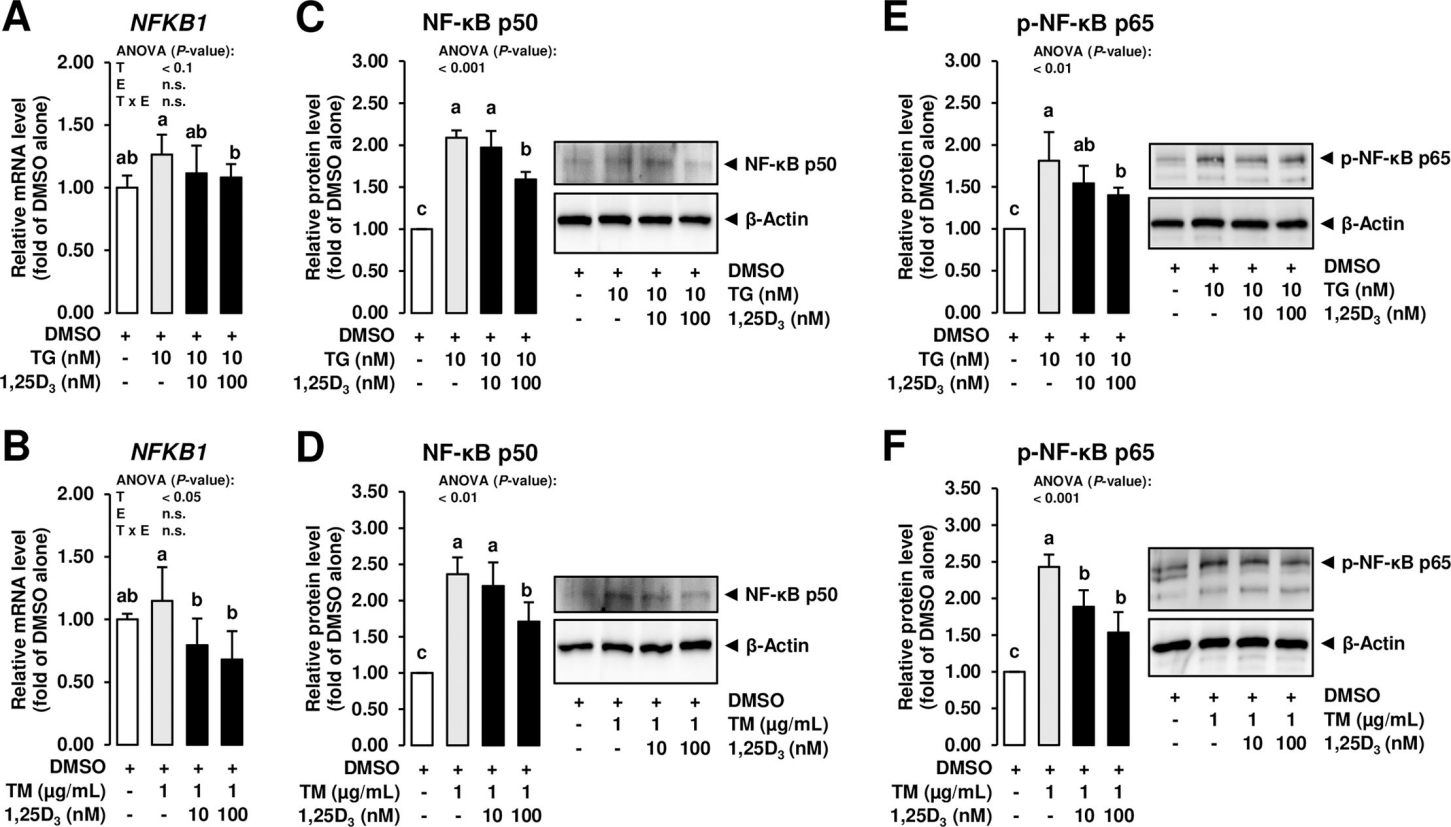
Effect of ER stress inducers alone and combined effect of ER stress inducers and 1,25D_3_ on expression of NF-κB in mammary epithelial cells. MCF-7 cells were pre-incubated in DMEM with 1% FBS and with either vehicle alone (DMSO, 0.1% v/v) or 1,25D_3_ (10 or 100 nM) alone for 24 h and subsequently co-incubated in DMEM with 1% FBS and with either vehicle alone (DMSO, 0.1% v/v), TG (10 nm; A, C, E) or TM (1 mg/mL; B, D, F) alone or 1,25D_3_ (10 or 100 nM) together with TG (10 nM; A, C, E) or TM (1 μg/mL; B, D, F) for additional 24 h. Bars represent relative mRNA levels (A, B) and relative protein levels (C-F) expressed as fold of vehicle alone and are means ± SD from three independent experiments. Bars with unlike letters are significantly different (*P* < 0.05). 2-factorial ANOVA classification factors: treatment (T), experiment (E), interaction (T x E). Abbreviations: NF-κB, nuclear factor-κB.

To finally study whether the inhibitory effect of 1,25D_3_ on ER stress induction affects the inflammatory response of MECs or not, the mRNA level of NF-κB p50 subunit (encoded by *NFKB1*) and the protein concentration of NF-κB p50 in nuclear extracts of MCF-7 cells was determined. NF-κB acts as the key regulator of the inflammatory process associated with mastitis development and is typically activated in the mammary gland epithelium *via* TLRs which sense specific PAMPs from pathogenic bacteria such as LPS. Upon activation of this transcription factor, a large set of genes encoding cytokines, chemokines, adhesion molecules and other pro-inflammatory products are induced and contribute to a pronounced burst of inflammatory mediator secretion from MECs [9–12]. Despite the rapid NF-κB-driven inflammatory response is important to effectively combat the infectious bacteria, the inflammatory process must be controlled to protect MECs from severe cellular damage and cell death, because production of milk components depends on the number and activity of vital MECs [51], whereas cellular death of MECs and parenchymal fibrosis after infection reduces the synthetic capacity of the mammary gland epithelium [52]. While treatment with both ER stress inducers increased the nuclear protein level of NF-κB p50 subunit compared to treatment with DMSO alone, the mRNA level of *NFKB1* and the protein level of NF-κB p50 subunit were decreased by the high concentration of 1,25(OH)_2_D_3_ compared to cells treated with either TG or TM alone (*P* < 0.05; **Fig 6A–6D**). While the major step in the initiation of NF-κB-dependent gene transcription involves phosphorylation, ubiquitination, and degradation of inhibitor of κB (IκB) proteins, which sequester NF-κB in the cytosol in resting cells, thereby allowing NF-κB to enter the nucleus where it can bind to regulatory sequences of target genes [53], it has been demonstrated that this step alone is often not sufficient to initiate gene expression. Inducible post-translational modification of NF-κB subunits by phosphorylation of multiple phosphor-acceptor sites has been shown to be equally important for initiation of NF-κB-dependent gene transcription [54]. In order to address this, the protein level of p-NF-κB p65 was determined in MCF-7 cells exposed to ER stress inducers and 1,25D_3_. Like unphosphorylated NF-κB, the protein level of p-NF-κB was increased by both ER stress inducers compared to treatment with DMSO alone, but decreased by the high concentration of 1,25D_3_ compared to cells treated with either TG or TM alone (*P* < 0.05; **Fig 6E and 6F**). These findings clearly indicated that the NF-κB-regulated inflammatory process induced by ER stress is inhibited by of 1,25D_3_ in MCF-7 cells. Considering that prolonged ER stress causes a persistent inflammatory process and leads to cell death *via* apoptosis, the observed inhibition of ER stress by 1,25D_3_ in MCF-7 cells can be interpreted as beneficial with regard to prevention and treatment of the inflammatory response associated with mastitis.

## Conclusion

Although a general limitation of this study is the use of a transformed human breast cancer cell line, which displays differences from normal MECs with regard to the abundance of certain receptors, such as estrogen receptors [55], and the response to non-physiological (e.g. exogenous retinoic acid) stimuli [56], both MCF-7 cells and normal MECs cells exhibit a similar regulation by important lactogenic hormones including oxytocin and prolactin [57–59]. In addition, MCF-7 cells like normal MECs are VDR positive cells and exposure to 1,25D_3_ causes a marked induction of *CYP24A1* and several immune response genes [37]. Moreover, cellular stress signaling, such as ER stress-induced activation of the UPR *via* the ER stress signaling proteins ATF6, IRE1 and PERK and ER stress-mediated activation of NF-κB, occurs largely identical in MCF-7 cells [60, 61] like in non-cancer MECs, thus, allowing to use the MCF-7 cell line specifically for studying the potential of 1,25D_3_ to modulate ER stress-induced NF-κB-driven inflammatory response in MECs. The present findings show that 1,25D_3_ is effective in attenuating ER stress and the NF-κB-driven inflammatory response in MCF-7 cells. This indicates that attenuation of ER stress by 1,25D_3_ in MECs may contribute to the recently observed inhibitory effect of intramammary treatment of dairy cows with 1,25D_3_ on the inflammatory process associated with mastitis [6–8]. In addition, the observation that the expression of *VDR* decreased upon induction of ER stress and 1,25D_3_ increased *VDR* expression in MCF-7 cells exposed to ER stress demonstrates that 1,25D_3_ counter-regulates the inhibitory effect of ER stress on *VDR* expression in MECs. Moreover, ER stress altered the expression of MEC hydroxylases involved in regulating 1,25D_3_ levels in a way which favors an increase of 1,25D_3_ levels, whereas 1,25D_3_ during ER stress modulated the expression of hydroxylases regulating 1,25D_3_ levels in a way which promotes a decrease of 1,25D_3_ levels. Albeit being speculative, it appears that the protective effect of 1,25D_3_ against ER stress in MCF-7 cells involves an improved responsiveness to 1,25D_3_ through induction of *VDR* expression, while stimulation of 1,25D_3_ production during ER stress may be interpreted as an adaptive response to the impaired responsiveness to 1,25D_3_ of MCF-7 cells exposed to ER stress.

## Supporting information

S1 Raw images (PDF)
