# Supplementary material for: 1,25-hydroxyvitamin D3 decreases endoplasmic reticulum stress-induced inflammatory response in mammary epithelial cells
Source: PLoS One. 2020 Feb 10;15(2):e0228945. doi: 10.1371/journal.pone.0228945 (PMC7010291; doi:10.1371/journal.pone.0228945)

S1_raw_images

Blots of Figure 2

HSPA5

L1	L2	L3	L4	L5	L6	L7	L8	L9
Marker					DMSO	DMSO	DMSO	DMSO
					-	10 nM TG	10 nM TG	10 nM TG
					-	-	10 nM 1,25(OH)2D3	100 nM 1,25(OH)2D3

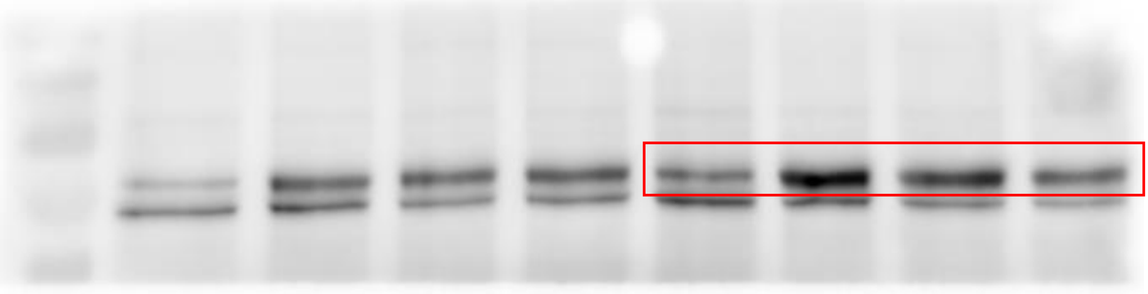

DDIT3

L1	L2	L3	L4	L5	L6	L7	L8	L9
Marker					DMSO	DMSO	DMSO	DMSO
					-	10 nM TG	10 nM TG	10 nM TG
					-	-	10 nM 1,25(OH)2D3	100 nM 1,25(OH)2D3

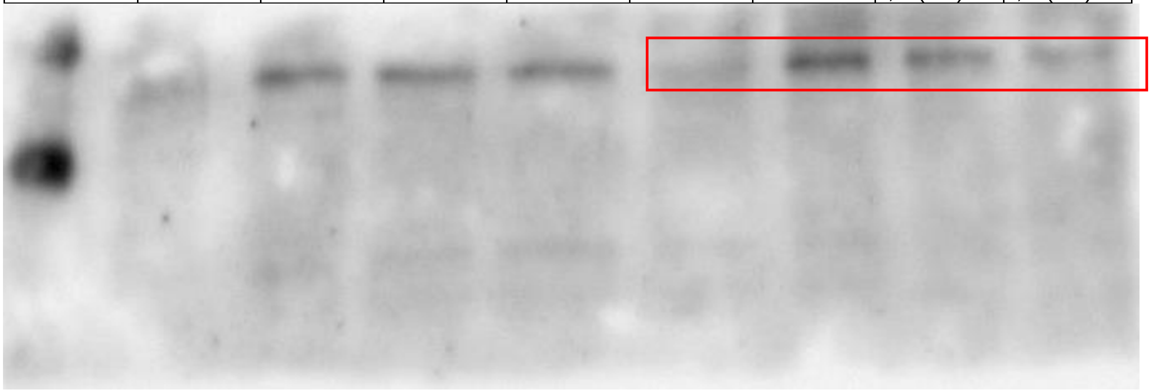

ß-Actin

L1	L2	L3	L4	L5	L6	L7	L8	L9
Marker					DMSO	DMSO	DMSO	DMSO
					-	10 nM TG	10 nM TG	10 nM TG
					-	-	10 nM 1,25(OH)2D3	100 nM 1,25(OH)2D3

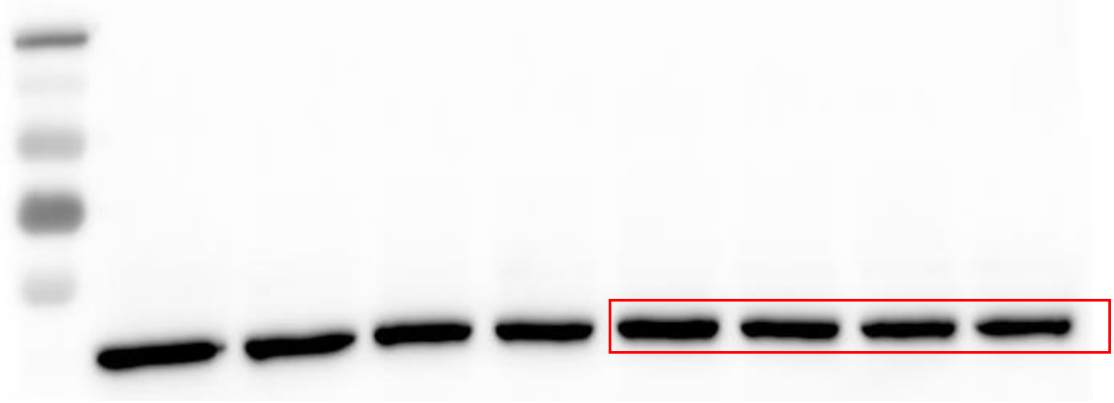

HSPA5

L1	L2	L3	L4	L5	L6	L7	L8	L9
Marker	DMSO	DMSO	DMSO	DMSO				
	-	10 µg/mL TM	10 µg/mL TM	10 µg/mL TM				
	-	-	10 nM 1,25(OH)2D3	100 nM 1,25(OH)2D3				

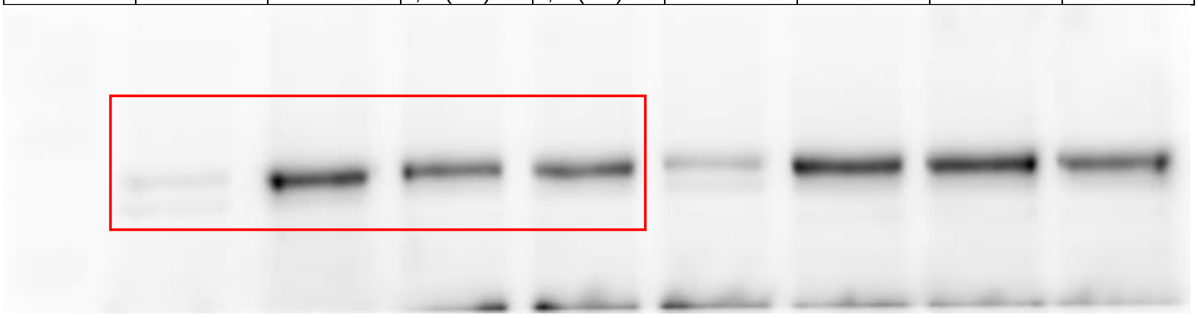

DDIT3

L1	L2	L3	L4	L5	L6	L7	L8	L9
Marker	DMSO	DMSO	DMSO	DMSO				
	-	10 µg/mL TM	10 µg/mL TM	10 µg/mL TM				
	-	-	10 nM 1,25(OH)2D3	100 nM 1,25(OH)2D3				

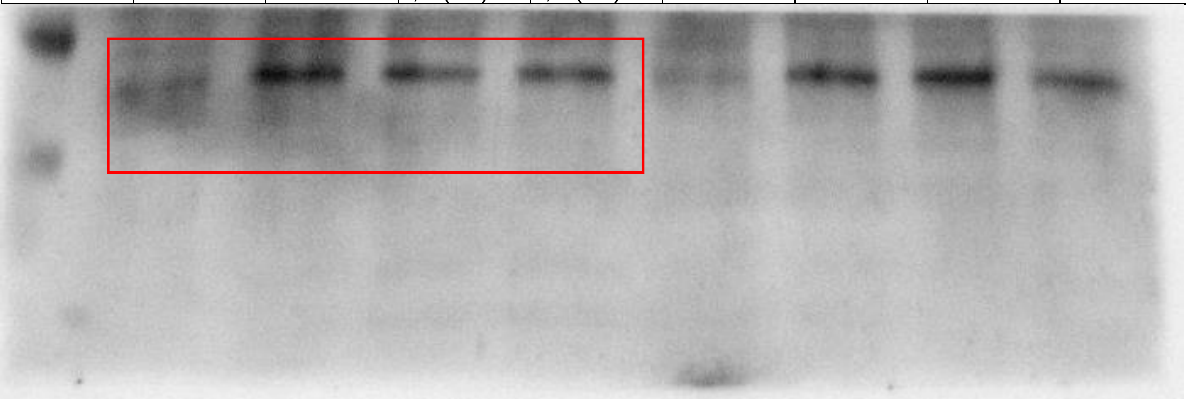

ß-Actin

L1	L2	L3	L4	L5	L6	L7	L8	L9
Marker	DMSO	DMSO	DMSO	DMSO				
	-	10 µg/mL TM	10 µg/mL TM	10 µg/mL TM				
	-	-	10 nM 1,25(OH)2D3	100 nM 1,25(OH)2D3				

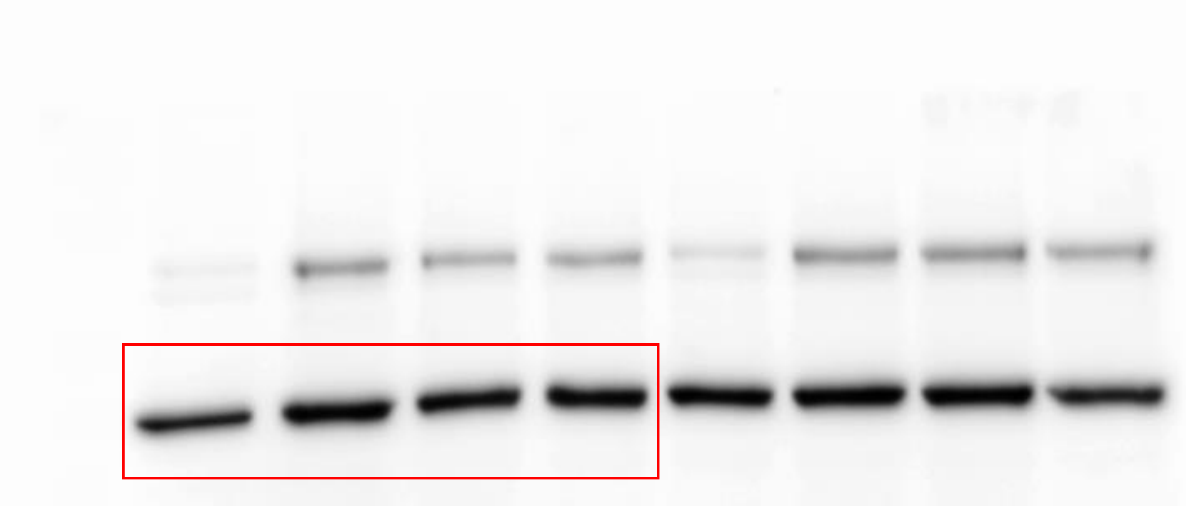

Blots of Figure 3

VDR

L1	L2	L3	L4	L5	L6	L7	L8	L9
Marker	DMSO	DMSO	DMSO	DMSO	DMSO	DMSO	DMSO	DMSO
	-	10 µg/mL TM	10 µg/mL TM	10 µg/mL TM	-	10 nM TG	10 nM TG	10 nM TG
	-	-	10 nM 1,25(OH)2D3	100 nM 1,25(OH)2D3	-	-	10 nM 1,25(OH)2D3	100 nM 1,25(OH)2D3

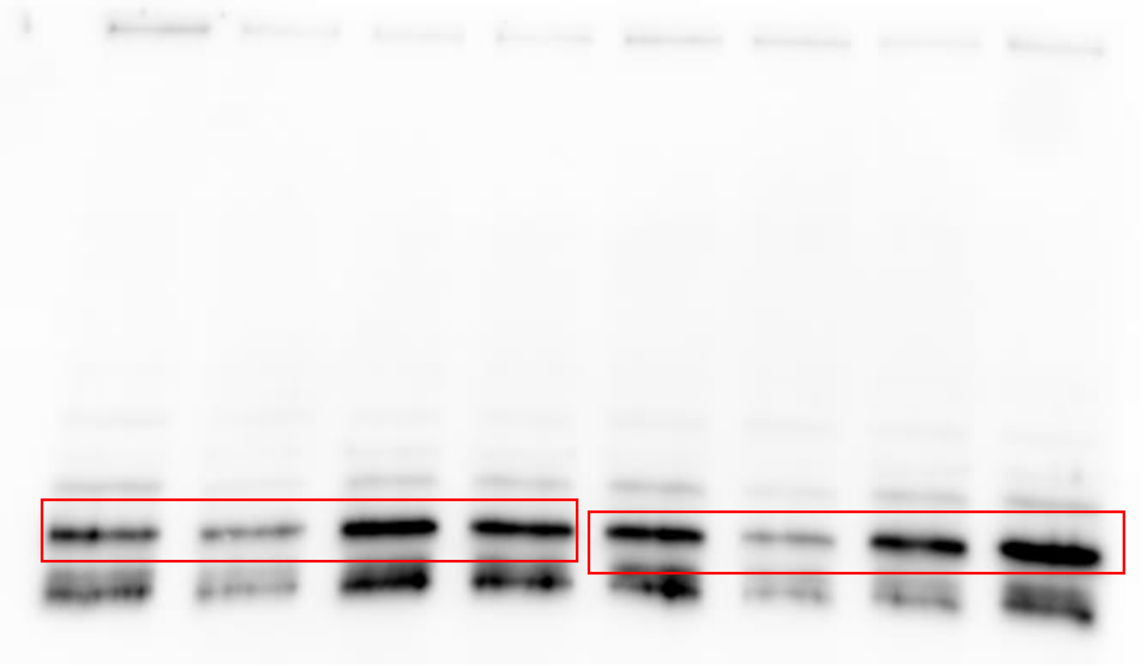

Vinculin

L1	L2	L3	L4	L5	L6	L7	L8	L9
Marker	DMSO	DMSO	DMSO	DMSO	DMSO	DMSO	DMSO	DMSO
	-	10 µg/mL TM	10 µg/mL TM	10 µg/mL TM	-	10 nM TG	10 nM TG	10 nM TG
	-	-	10 nM 1,25(OH)2D3	100 nM 1,25(OH)2D3	-	-	10 nM 1,25(OH)2D3	100 nM 1,25(OH)2D3

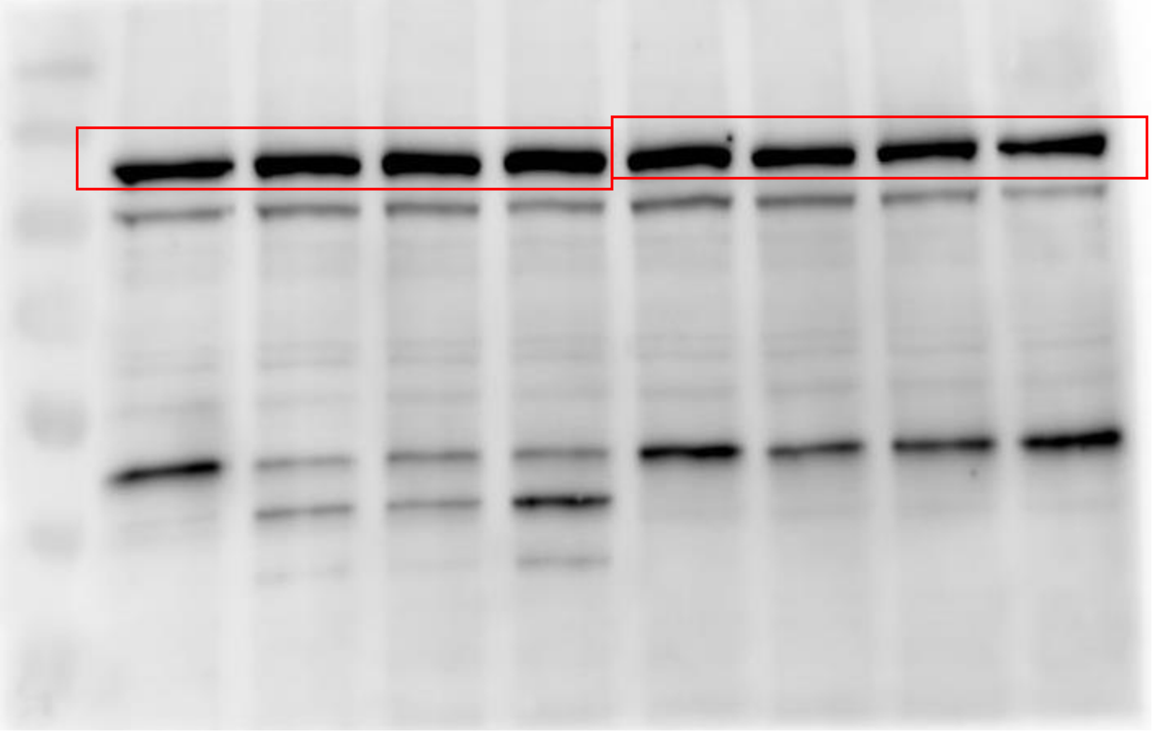

Blots of Figure 6

NF-kB

L1	L2	L3	L4	L5	L6	L7	L8	L9
Marker					DMSO	DMSO	DMSO	DMSO
					-	10 nM TG	10 nM TG	10 nM TG
					-	-	10 nM 1,25(OH)2D3	100 nM 1,25(OH)2D3

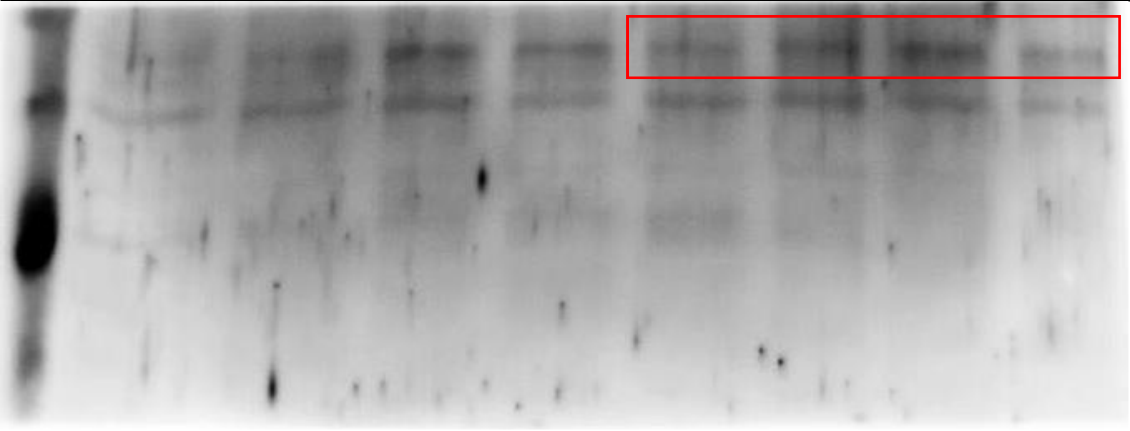

ß-Actin

L1	L2	L3	L4	L5	L6	L7	L8	L9
Marker					DMSO	DMSO	DMSO	DMSO
					-	10 nM TG	10 nM TG	10 nM TG
					-	-	10 nM 1,25(OH)2D3	100 nM 1,25(OH)2D3

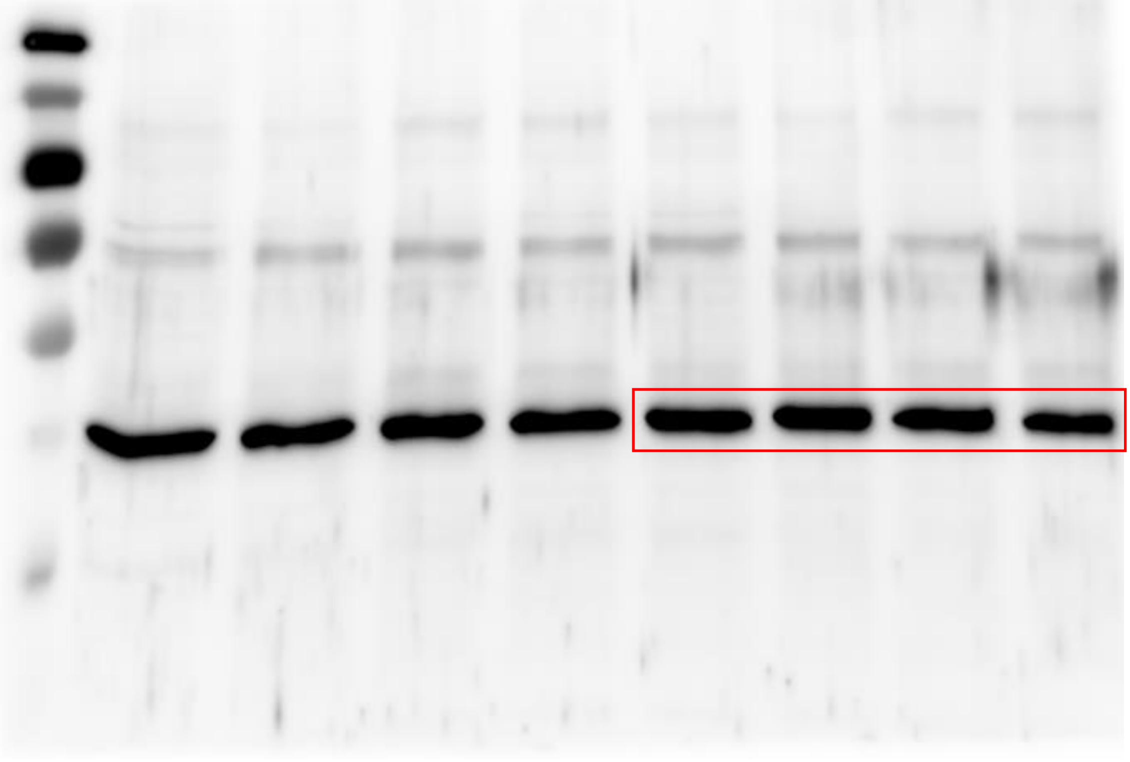

NF-kB

L1	L2	L3	L4	L5	L6	L7	L8	L9
Marker	DMSO	DMSO	DMSO	DMSO				
	-	10 µg/mL TM	10 µg/mL TM	10 µg/mL TM				
	-	-	10 nM 1,25(OH)2D3	100 nM 1,25(OH)2D3				

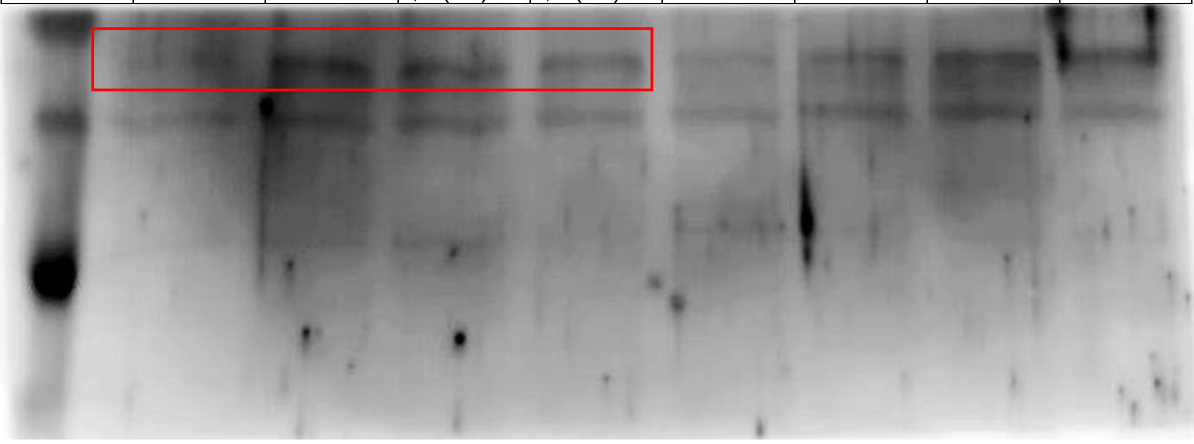

ß-Actin

L1	L2	L3	L4	L5	L6	L7	L8	L9
Marker	DMSO	DMSO	DMSO	DMSO				
	-	10 µg/mL TM	10 µg/mL TM	10 µg/mL TM				
	-	-	10 nM 1,25(OH)2D3	100 nM 1,25(OH)2D3				

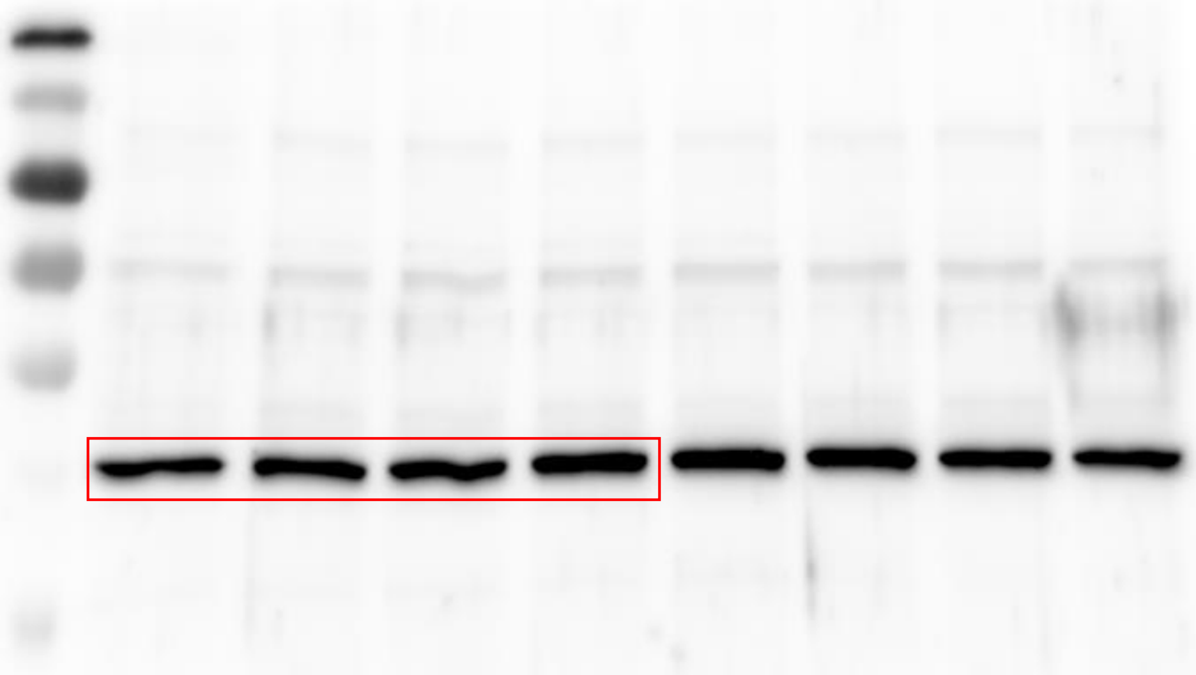

p-NF-kB

L1	L2	L3	L4	L5	L6	L7	L8	L9
Marker	DMSO	DMSO	DMSO	DMSO				
	-	10 µg/mL TM	10 µg/mL TM	10 µg/mL TM				
	-	-	10 nM 1,25(OH)2D3	100 nM 1,25(OH)2D3				

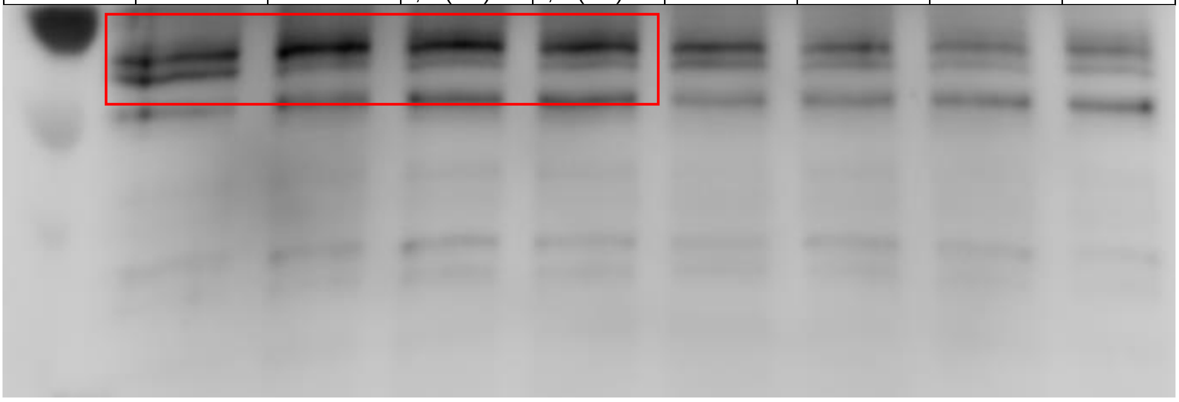

ß-Actin

L1	L2	L3	L4	L5	L6	L7	L8	L9
Marker	DMSO	DMSO	DMSO	DMSO				
	-	10 µg/mL TM	10 µg/mL TM	10 µg/mL TM				
	-	-	10 nM 1,25(OH)2D3	100 nM 1,25(OH)2D3				

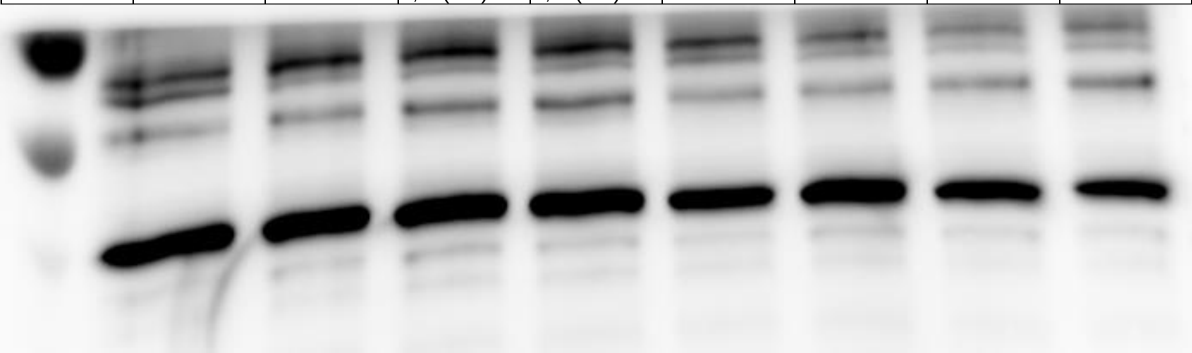

p-NF-kB

L1	L2	L3	L4	L5	L6	L7	L8	L9
Marker					DMSO	DMSO	DMSO	DMSO
					-	10 nM TG	10 nM TG	10 nM TG
					-	-	10 nM 1,25(OH)2D3	100 nM 1,25(OH)2D 3

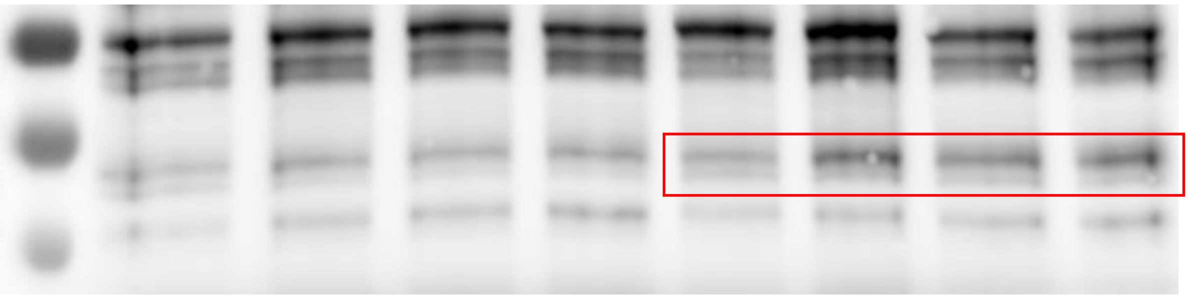

ß-Actin

L1	L2	L3	L4	L5	L6	L7	L8	L9
Marker					DMSO	DMSO	DMSO	DMSO
					-	10 nM TG	10 nM TG	10 nM TG
					-	-	10 nM 1,25(OH)2D3	100 nM 1,25(OH)2D3

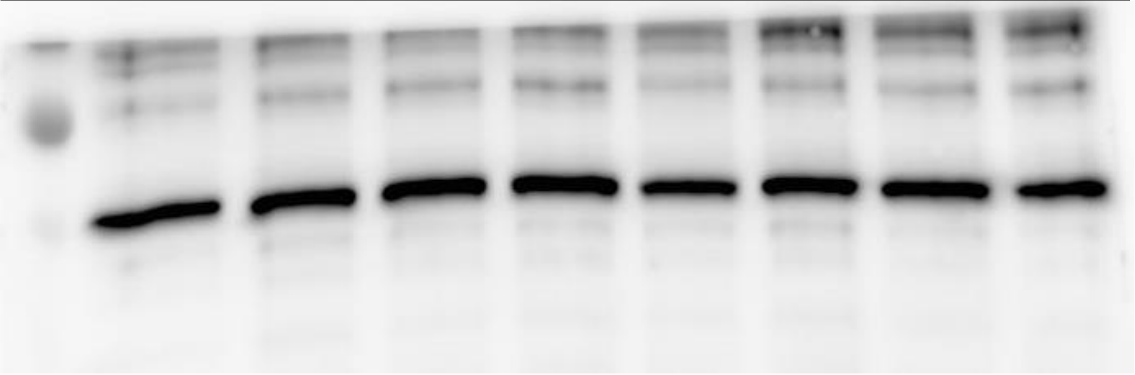

Supplement: S1 Raw images — (PDF) [file pone.0228945.s001.pdf]
